# Descriptive Analysis of Typhoid Fever Surveillance Data in the Jimma Zone, Southwest Ethiopia (2015–2019)

**DOI:** 10.1155/2021/1255187

**Published:** 2021-12-13

**Authors:** Getaneh Atikilt Yemata, Chalachew Yenew, Melkalem Mamuye, Mulu Tiruneh, Tigabnesh Assfaw, Sileshi Mulatu, Ermias Sisay, Fitalew Tadele

**Affiliations:** ^1^Debretabor University, College of Health Sciences, Public Health Department, Debre Tabor, Ethiopia; ^2^Bahir Dar University, College of Medicine and Health Sciences, Department of Pediatrics and Child Health Nursing, Bahir Dar, Ethiopia; ^3^Debretabor University, College of Health Sciences, Department of Pediatrics and Child Health Nursing, Debre Tabor, Ethiopia; ^4^Debretabor University, College of Health Sciences, Biomedical Science Department, Debre Tabor, Ethiopia

## Abstract

**Introduction:**

Typhoid fever is a major cause of morbidity and mortality around the globe, and it is a serious illness in developing countries. Typhoid fever is prevalent in Ethiopia, and the burden differs with diverse demography, environment, and climate. The study aimed to determine the incidence of typhoid fever cases by person, place, and time.

**Method:**

A descriptive cross-sectional study was conducted among the five years (2015–2019) of surveillance data of typhoid fever in the Jimma Zone, Oromia Region, Ethiopia. The data were extracted from the zonal health management information system database from May to June 2020. SPSS version 21 was used to enter and analyze the data. Descriptive analysis was used to assess the distribution of typhoid fever incidence in time, place, and personal groups.

**Result:**

A total of 36,641 individuals suffered from typhoid fever during the five years. Among these, 18,972 (51.8%) were females and 17,669 (48.2%) were males. Incidence of typhoid fever was found as follows: 216, 198, 203, 264, and 299 cases per 100,000 persons were reported during 2015, 2016, 2017, 2018, and 2019, respectively. Typhoid fever cases were increased by 1.4 from 2015–2019. A high incidence of cases was observed at the start of wet months. The majority of the investigated cases were identified in Kersa, 4,476 (12.2%), Gomma, 4,075 (11.1%), and Mana, 3,267 (8.9%), woredas. Of the total, 151 (0.4%) of the reported cases were admitted for inpatient care. During the five years of surveillance data, death was not reported from all woredas. *Conclusion and Recommendation*. Typhoid fever was a major public health problem in the Jimma Zone for the last 5 years, and it was increased through the years. Zonal health departments should strengthen the interventions focused on the woredas that had a high burden of typhoid fever at the start of the wet months.

## 1. Introduction

Typhoid fever is a major cause of morbidity and mortality around the globe, and it is a serious illness in developing countries. Reports that estimate the global burden of typhoid fever indicate that the disease is still a public health issue. Annually in the globe, there are around 14.3 million typhoid fever cases leading to 135.9 thousand deaths and 9.8 million DALYs. In developing countries, around 12.5 million persons are affected by typhoid fever each year [1, 2].

Typhoid fever is one of the public health significant diseases in low-income countries such as Ethiopia in which the safety of drinking water supply, hygienic condition, and quality of life are poor and below standard. If left untreated, typhoid fever is a lethal disease where 10–30% of cases will end in death, while with intervention, the case fatality rate will be dropped to 1–4% [3].

In Ethiopia, similar to other low-income countries, there was a challenge to evaluate and determine the burden of typhoid fever cases due to inadequate coverage of studies and a dearth of coordinated surveillance systems in the country. Besides, a low report rate of positive cases and the occurrence of other infectious diseases that gets high importance in the surveillance system may make the problem of typhoid fever appear small by comparison and lack concern [4, 5].

According to the Ethiopia Federal Ministry of Health (FMOH) report, two percent of males and three percent of females died because of typhoid fever [6]. Among the symptomatic cases of typhoid fever, around 10–40% will have a risk of complication and death if left untreated. Even from typhoid fever patients who had got treatment, two to five percent will have a risk of death. In Ethiopia, typhoid fever is an endemic- and epidemic-prone disease, especially in the area of highlands in which around 10,000 cases are reported each year. People without a home who live in together with poor hygienic conditions are primarily affected by the disease [7].

Thus far, the FMOH has established different intervention strategies for the community to improve awareness and risk perception of infectious diseases including typhoid fever through health extension workers. However, the burden of typhoid fever has not been reduced to an acceptable rate [6].

Bearing in mind, in Ethiopia, typhoid fever is an endemic disease, and the burden and consequence of the disease vary with different ecology, climate, and population groups. So, communicating the latest new information concerning the distribution of typhoid fever in the catchment area will have valuable significance for early and prioritized interventions [8].

Typhoid fever is most common among individuals who work in food handling and preparation activities and overcrowding slums and low-economic-status people because of poor hygiene and waste disposal system. Unless protective and control measures are taken appropriately, the occurrence of typhoid fever outbreaks would be higher [9].

A continuous investigation of surveillance data is significant for noticing disease outbreaks, judging disease tendencies, and evaluating the efficacy of disease prevention and control programs. In addition, the surveillance data analysis provides indicative information about the appropriate health resource allocation [10]. Despite the presence of collected data in the zonal health departments, it is not analyzed by considering the epidemiology of disease distribution which it helps to know the at-risk population, trends over time, and the geographical distribution of the diseases, which in turn helps for appropriate intervention [11]. Therefore, this study aimed to assess the incidence and mortality rate of typhoid fever by person, place, and time among the five years (2015–2019) of surveillance data in the Jimma Zone.

## 2. Methods

### 2.1. Study Area

**Figure 1:**
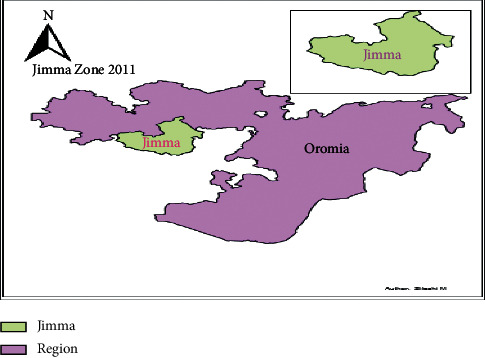
Map of the Jimma Zone in the Oromia Region, Ethiopia [13].

The surveillance data analysis was employed in the Jimma Zone, which is one of the 20 rural zones in the Oromia Region, located in the southwest of Ethiopia with a distance of 352 km from Addis Ababa. The total area of the zone is 199,316.18 km^2^, accounting for about 17.2% of the total area of the region (Figure 1). Climatically, the zone is classified into three agroecological zones, namely, highlands (14.9%), midlands (21.5%), and lowlands (63.5%). The annual rainfall ranges from 900 ml to 1,400 ml. The annual mean temperature of the zone ranges from 10°C to 25°C, with a mean temperature of 17.5°C. Fifty-three percent of the zone is exposed to malaria, which is a febrile illness that shares similar symptomatology and epidemiology to typhoid fever. Both share similar social circumstances that require attention for their transmission [12].

Administratively, the zone is divided into 21 administrative woredas (19 rural woredas and 2 towns) and 566 kebeles. According to the Ethiopian 2011 population and housing census projection, the total population of the zone in July 2019 was estimated to be 3,425,206 with 1,678,351 males and 1,746,855 females. The average household size in the zone is 4.8, with a population density of 32.1, which greatly varies among zones and woredas. Likewise, the Jimma Zone has 5 hospitals, 122 health centers, 566 health posts, 10 private medium clinics, 101 lower clinics, 31 drug stores, and 41 rural drug vendors.

### 2.2. Study Period

The study was conducted among the monthly report of 5-year (January 2015–December 2019) typhoid fever surveillance data, and it was extracted from May to June 2020.

### 2.3. Study Design

A descriptive cross-sectional study design was employed to assess the distribution of typhoid fever incidence in a different time, person, and place from the five-year (2015–2019) facility-based (sentinel) surveillance data of typhoid fever in the Jimma Zone.

### 2.4. Population

The source populations were all people at risk of typhoid fever in the Jimma Zone, Oromia Region. All the population of the Jimma Zone that visits the health institution under surveillance in the zone and diagnosed with typhoid fever during 2015–2019 was the population of the study.

### 2.5. Eligibility Criteria

Individuals who visit health facilities under surveillance systems in the Jimma Zone during 2015–2019 were included in the study. Individuals suspected of typhoid fever but with no laboratory confirmation or incomplete record of the outcome variable in the database were excluded from the study.

## 3. Study Variable

### 3.1. Incidence of Typhoid Fever

#### 3.1.1. Case Definitions of Typhoid Fever

Suspected: a person presented with gradual onset of remittent fever or fever rising in a stepladder fashion in the first week and manifested with arthralgia, loss of appetite, headache, abdominal pain, and constipation or diarrhea. Confirmed: a suspected case with the Widal test, “O” titer of 1/160 and more, and very suggestive of recent infection of typhoid fever or a suspected case with positive blood culture at the 1st week or positive stool culture at the 3rd, 4th, and 5th week of illness which is very definitive for typhoid fever [1].

Report completeness: the proportion of all expected Integrated Disease Surveillance report (IDSR) summary reports in the district or zone that are submitted to the zonal HMIS unit [14].

#### 3.1.2. Data Collection Method

Data used for the study were based on the records of the health office of the Jimma Zone municipality. All health facilities under surveillance systems in the Jimma Zone were included in the study. Two experienced field epidemiology professionals extracted data on all typhoid fever cases during the five consecutive years from the zonal health office Health Management Information System (HMIS) databases through a document review checklist. The checklist was adapted from the WHO [3], and its elements included the date of report, age, sex, admission hospitalization, laboratory finding, and final case classification. All typhoid fever cases from 2015–2019 were extracted and used for the study.

#### 3.1.3. Data Processing and Analysis

Data were entered in EpiData version 3.1 and exported to SPSS version 21 for data cleaning and analysis. The data were analyzed and interpreted from July–September 2020. Incorrect records and errors were cleaned before analysis. Descriptive analysis was used to determine the distribution of typhoid fever incidence in different times (months and years), woredas, and personal groups (sex and age category). Incidence rate, percent of cases, and report completeness were computed to describe the results. Typhoid fever incidence was calculated by taking the total number of new cases of typhoid fever and dividing that by the sum of the person-time of the at-risk population in the study area from 2015–2019. Report completeness in this study was measured as the number of reports received from facilities (monthly) divided by the expected number of reports from facilities [14, 15]. Textual narration, tables, and graphs were used to describe the results. Finally, the findings of our study were reported as frequency, percentage, and rates.

## 4. Result

### 4.1. Distribution of Typhoid Fever by Person

**Figure 2:**
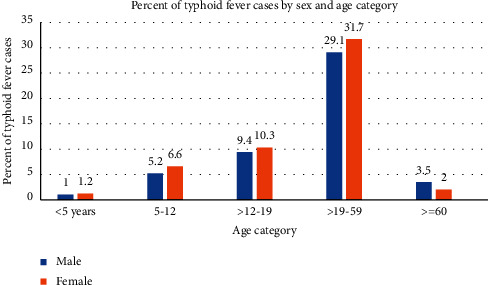
Distribution of typhoid fever cases by sex and age category in the Jimma Zone, Oromia Region, Southwest Ethiopia, from 2015 to 2019.

All of the surveillance data which were recorded from January 2015 to December 2019 that fulfill the eligibility criteria were included in the study. A total of 36,641 typhoid fever cases were registered from 2015 to 2019. Among these, 18,972 (51.8%) were females and 17,669 (48.2%) were males. The highest number of typhoid fever cases was observed among study subjects in the age category of 19–59 years, 22,300 (60.8%) (Figure 2).

The age category was classified for comparison according to previous studies and reports [4, 16].

### 4.2. Zonal Disease Trends

#### 4.2.1. Incidence of Typhoid Fever by Time

In the last 5 years (2015 to 2019), the zonal PHEM unit received 36,641 reports concerning cases of typhoid fever in the Jimma Zone. The median number of cases was 6,265 per year, and the highest number of cases (9,899) was recorded in 2019 (Table 1).

**Figure 3:**
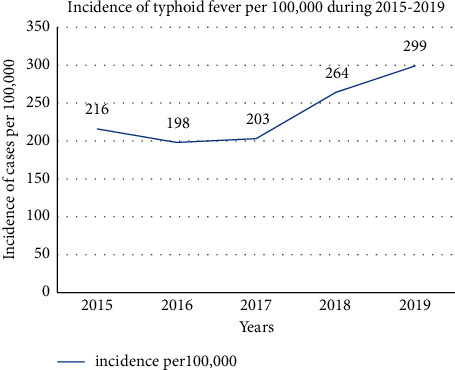
Distribution of typhoid fever incidence by year in the Jimma Zone, Oromia Region, Southwest Ethiopia, from 2015–2019.

The uppermost annual incidence rate (299 per 100,000) of typhoid fever was reported during 2019. The annual incidence of typhoid fever was increased from 216 to 299 per 100,000 persons by 1.4 in 2015–2019. We estimated the 2015–2019 annual incidence of typhoid fever in the Jimma Zone to be 238 per 100,000 person-year. According to the reported surveillance data, there was no death due to typhoid fever during the five consecutive years (Figure 3).

#### 4.2.2. Distribution of Morbidity by Months vs. Years

**Figure 4:**
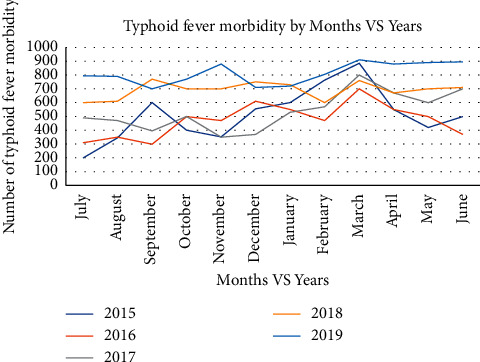
Trends of typhoid fever by months in the Jimma Zone, Oromia Region, Southwest Ethiopia, from 2015 to 2019.

From a total of typhoid fever cases (36,641) during the five years, there was a report in all months. The highest number of cases (910) was reported during March 2019. The highest number of cases (peak) per month in each year was recorded as in 2015, it was in March and September, in 2016, during March and December, in 2017, during March, June, and September, in 2018, during November, June, and August, and in 2019, during October, March, and June. There was a constantly increasing number of cases from November to March in 2015, 2017, and 2019. Furthermore, there was a small peak in all years during March, except in 2018. However, there was no constant peak time throughout the 5 years. Overall, the incidence was not uniform, but it was on an increasing mode in some months, and September, November, October, March, and June were the months in which the highest number of cases was recorded (Figure 4).

#### 4.2.3. Distribution of Typhoid Fever in Place or Woredas

**Figure 5:**
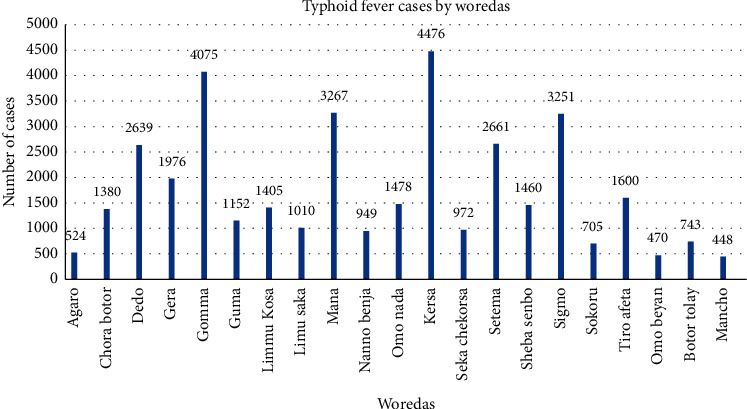
Typhoid fever cases by woredas in the Jimma Zone, Oromia Region, Southwest Ethiopia, from 2015 to 2019.

When we see the distribution of typhoid fever cases by woredas during the five years (2015–2019), the zonal PHEM unit received surveillance reports from 21 woreda health offices and 5 hospitals. From the total report of 36,641 typhoid fever cases, the majority of the report was from Kersa woreda, 4,476 (12.2%), Gomma woreda, 4,075 (11.1%), and Mana woreda, 3,267 (8.9%). Moreover, all hospitals reported the lowest cases, which is 0% to 1% within the last five years (Figure 5).

#### 4.2.4. Distribution of Morbidity by Woredas/Places

**Figure 6:**
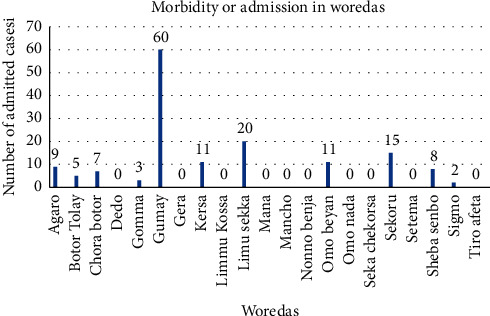
Distribution of typhoid fever morbidity on admission by woredas of the Jimma Zone, Oromia Region, Southwest Ethiopia.

A total of 151 (0.4%) cases were admitted or hospitalized due to typhoid fever. Of these admitted cases, the majority were reported from Gumay woreda 60 (39.7%), and around ten woredas have no admission of typhoid fever. Furthermore, no mortality occurred at all woredas (Figure 6).

#### 4.2.5. Number of Health Facilities in the Jimma Zone during 2015–2019

**Figure 7:**
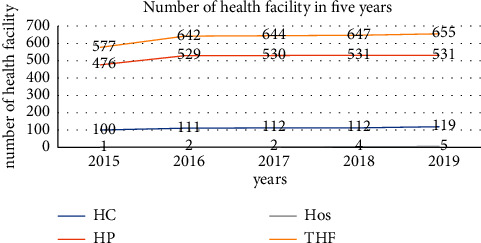
Number of health facilities expected to be reported by year in the Jimma Zone, Oromia Region, from 2015 to 2019.

In the Jimma zone, during 2015, the Total number of Health Facilities (THF) was 577, which include 476 Health Posts (HPs), 100 Health Centers (HCs), and 1 Hospital (Hos). After five years, in 2019, the number of health institutions was increased to 531 health posts, 119 health centers, and 5 hospitals, and the total number of health facilities was 655 (Figure 7). The number of health facilities was increased through the five consecutive years. Besides, the health facility coverage was increased by 90% in 2019 as compared to 15% in 2015.

#### 4.2.6. Report Completeness

Facility report completeness in the Jimma Zone was increased from 2015 to 2019 by 3%. The percentage of report completeness of the zone (from all types of health facilities) in 2015 and 2019 was 95% and 98%, respectively (Table 2).

## 5. Discussion

This study assessed the incidence of typhoid fever by person, place, and time among the five years (2015–2019) of surveillance data in the Jimma Zone with a good quality of the report.

The total incidence of typhoid fever cases in the five years (2015–2019) was 238 per 100,000 persons. The incidence of typhoid fever in each year was investigated as 216 cases in 2015, 198 cases in 2016, 203 cases in 2017, 264 cases in 2018, and 299 cases in 2019 per 100,000 persons. The result suggested that the burden of typhoid fever cases was increased from 2015 to 2019 with a fluctuating trend, even though different interventions have been implemented in the catchment area. The trend is congruent with a study in Lalo Asabi district, West Wollega, Ethiopia, which was increasing in an instable mode [17]. It could be due to the expansion of hospitals and health centers and health service coverage through the consecutive years in the Jimma Zone, which can investigate an increased number of typhoid fever cases.

The result of our study was congruent to the national health and health-related indicator report in the Oromia region and at the national level [18], and it was in line with a study conducted in Africa (Malawi and Seralione) [19], while the findings of this study were lower as compared to a study conducted in Shashemene Hospital and Afar District, Ethiopia [8, 11]. The discrepancy could be due to study setting, sample size, and target population difference. The attempt of the healthcare providers including health extension workers in the area or the validity of a diagnostic test could contribute to the variations.

This result suggested that the magnitude of typhoid fever rises at the start of wet months (end of the dry season) since the highest burden was observed during March. In general, September, October, November, March, and June encountered a high number of typhoid fever cases. Even though typhoid fever cases are detected throughout the year, in endemic areas, the high incidence is mostly reported during summer or wet months [20]. In addition, the seasonality of typhoid fever is supported by a scientific report which argued that, during wet months, a preceding peak of rainfall is associated with the high incidence of typhoid fever cases [21]. The change in timing of rainy seasons was experienced in most parts of the country in which mid-March up to June was the main rainy season and the second rainy season shifted to September to mid-December in Southwestern Ethiopia during recent years [4]. In our study area context, the end of the dry season (the starting of wet months) is the time in which the rural water supply is the lowest and people congregate at the source of water. This may be the start of the rain month, and people living in the rural area utilize runoff water or pond water for drinking and cooking food without water treatment. At this time, the rain helps spread already contaminated water supplies [7]. Therefore, the opportunity of contamination of water with pathogenic organisms is high with the aid of poor sanitation practice [5].

The result of this study, concerning the distribution of typhoid fever cases in geographical areas or woredas in the Jimma Zone, concluded that the majority of the investigated cases were identified in Kersa, Gomma, Mana, Sigma, Setema, and Deedo woredas. Typhoid fever is more common in areas with poor sanitation practices [3], so unsafe and inadequate water for drinking and handwashing, especially related to the toilet, could be responsible for the increment of cases in the identified sites. This could generate the chance of transmission of the pathogen from one person to another person.

In our study, the admission rate in the five consecutive years was 0.4%, and most of the admitted cases were reported in Gumay woreda (39.7%), even though the incidence of typhoid fever cases in this woreda was lower (3.1%) as compared to that in other woredas. The case fatality rate was zero within 5 years. The morbidity and mortality of typhoid fever in the catchment area were minimal. This could be explained through the increasing awareness and health-seeking behavior, early detection of cases, appropriate diagnosis, and treatment that contribute to the minimization of inpatient cases and the absence of death.

Concerning the distribution of typhoid fever in sex, the result of this study showed that females accounted for 51.8% of cases, which is congruent as we compare to a gender-based analysis of the morbidity report of FMOH EFY, 52% of females encountered the cases, and in the Arba Minch Health Center, 52.3% of typhoid fever cases were females [4, 22]. Most (60.8%) of the study subjects that have been affected with typhoid fever were in the age category of above 19 years to 59 years, followed by 13–19 years. On the other side, under-five children and elderly (60 years and above) age groups were less affected. Our result is congruent with a study employed in the Arba Minch Health Center, Southern Ethiopia, which showed that study subjects with the age of 5–15 and 16–30 years were highly affected as compared to study subjects of age less than five years [4]. In addition, a study conducted in Jigjiga, Eastern Ethiopia, showed that the age group of 31–45 was more at risk to have typhoid fever [23]. A five-year study in Hohoe Municipality of the Volta Region, Ghana, argued that adults in the age group of 25–29 years were most affected with typhoid fever [24]. This indicated that adults and adolescent age groups were more vulnerable to typhoid fever infection. However, it was different from other studies which argued that children were more vulnerable to typhoid fever cases [25–27]. This could be due to the proportion of study participants in each age group, geographical location, and others.

This study examined typhoid fever incidence indicator data reporting completeness in the Jimma zone health management information system between 2015 and 2019, and it was greater than the standards of the national target (80%) [3]. The study concluded that it has good quality to offer an assessment of typhoid fever incidence.

### 5.1. Limitation

Most of the private clinics were not included in this surveillance data analysis because they were not in the government reporting system. In addition, there could be individuals who do not visit health facilities to get care. So, this study might not address all possible typhoid fever cases in the study area. The quality of surveillance data, particularly the consistency, could be affected through the fast expansion of health facilities over time. This implied that the increase of cases over time may not truly indicate the more occurrence of new cases over time; rather, there was a probability to be due to the increase of health facilities or increase of reporting sites.

## 6. Conclusions

Typhoid fever has been an important public health issue in the Jimma Zone for the last 5 years, and the increment of typhoid fever case incidence throughout the years was reported. In general, the majority of typhoid fever cases were treated at the outpatient department and few cases were treated at the inpatient department. The highest incidence of typhoid fever cases was reported at the start of wet months, particularly in March, and the distribution of cases was positively skewed to Qarsa, Gomma, and Mana woredas among the surveillance areas. Besides, adults were more vulnerable to typhoid fever.

### 6.1. Recommendation

This study aims to show the incidence and distribution of typhoid fever in different woredas of the Jimma Zone during 2015–2019 and offer evidence for the appropriate management and decision on the strategy for concerned bodies, particularly local health system administrators. Communicating the findings of this study to the governmental and other concerned bodies has valuable significance for early and prioritized interventions. So, designing appropriate interventions based on this study's findings and other necessary pieces of evidence has a direct impact on the reduction of typhoid fever occurrence and its consequences in the target population.

To avert the burden and consequences of the disease, healthcare providers should deliver intensive health promotion programs such as safe hygienic conditions, particularly handwashing related to cooking, eating, and toilet. Zonal health departments should strengthen the interventions focused on the woredas which had a high burden of typhoid fever at the start of wet months. Private health facilities or clinics must be included in the surveillance reporting system.

## Figures and Tables

**Table 1:** Incidence of typhoid fever by year in the Jimma Zone, Oromia Region, Southwest Ethiopia, from 2015 to 2019.

Years	Population at risk	Number of cases	Number of deaths	Percentage of cases^a^ (%)	Incidence rate (case/100,000)
2015	2,863,762	6,182	0	17	216
2016	2,976,050	5,874	0	16	198
2017	3,088,338	6,265	0	17	203
2018	3,200,626	8,421	0	23	264
2019	3,312,914	9,899	0	27	299
Total	15,441,690 person-year	36,641			238

^a^Percentage of cases: the number of typhoid fever cases in each year divided by the total number of typhoid fever cases of the five years.

**Table 2:** Zonal surveillance report (from all health facilities) completeness by year in the Jimma Zone, Oromia Region, from 2015 to 2019.

Years	Expected report^a^	Actual report^b^	Percentage of report completeness (%)
2015	577	521	95
2016	642	581	95
2017	644	542	97
2018	647	545	98
2019	655	625	98

^a^Expected report: total number of health facilities expected to be reported on the district HMIS in the Jimma Zone. ^b^Actual report: number of health facilities that submitted a report on the district HMIS in the Jimma Zone with a complete report of key data series.

## Data Availability

The data are prepared by considering the publishers research data policy and accessible on reasonable request.

## Abbreviations

EFY: Especially for youth
FMOH: Federal Ministry of Health
HMIS: Health Management Information System
IDSR: Integrated Disease Surveillance Report
PHEM: Public Health Emergency Management
WHO: World Health Organization.
